# Bioinformatic identification of *Mycobacterium tuberculosis* proteins likely to target host cell mitochondria: virulence factors?

**DOI:** 10.1186/2042-5783-2-9

**Published:** 2012-12-22

**Authors:** María Maximina Bertha Moreno-Altamirano, Iris Selene Paredes-González, Clara Espitia, Mauricio Santiago-Maldonado, Rogelio Hernández-Pando, Francisco Javier Sánchez-García

**Affiliations:** 1 Laboratorio de Inmunorregulación, Departamento de Inmunología, Escuela Nacional de Ciencias Biológicas, Instituto Politécnico Nacional, Carpio y Plan de Ayala, Col. Sto. Tomás, México D.F, México; 2 Departamento de Inmunología, Instituto de Investigaciones Biomédicas, Universidad Nacional Autónoma de México, México D.F, México; 3 Instituto Nacional de Ciencias Médicas y de la Salud “Salvador Zubirán”, México D.F, México

**Keywords:** *Mycobacterium tuberculosis*, Mitochondrial targeting, Virulence

## Abstract

**Background:**

*M. tuberculosis* infection either induces or inhibits host cell death, depending on the bacterial strain and the cell microenvironment. There is evidence suggesting a role for mitochondria in these processes.

On the other hand, it has been shown that several bacterial proteins are able to target mitochondria, playing a critical role in bacterial pathogenesis and modulation of cell death. However, mycobacteria–derived proteins able to target host cell mitochondria are less studied.

**Results:**

A bioinformaic analysis based on available genomic sequences of the common laboratory virulent reference strain *Mycobacterium tuberculosis* H37Rv, the avirulent strain H37Ra, the clinical isolate CDC1551, and *M. bovis* BCG Pasteur strain 1173P2, as well as of suitable bioinformatic tools (MitoProt II, PSORT II, and SignalP) for the *in silico* search for proteins likely to be secreted by mycobacteria that could target host cell mitochondria, showed that at least 19 *M. tuberculosis* proteins could possibly target host cell mitochondria. We experimentally tested this bioinformatic prediction on four *M. tuberculosis* recombinant proteins chosen from this list of 19 proteins (p27, PE_PGRS1, PE_PGRS33, and MT_1866). Confocal microscopy analyses showed that p27, and PE_PGRS33 proteins colocalize with mitochondria.

**Conclusions:**

Based on the bioinformatic analysis of whole *M. tuberculosis* genome sequences, we propose that at least 19 out of 4,246 *M. tuberculosis* predicted proteins would be able to target host cell mitochondria and, in turn, control mitochondrial physiology. Interestingly, such a list of 19 proteins includes five members of a mycobacteria specific family of proteins (PE/PE_PGRS) thought to be virulence factors, and p27, a well known virulence factor. P27, and PE_PGRS33 proteins experimentally showed to target mitochondria in J774 cells. Our results suggest a link between mitochondrial targeting of *M. tuberculosis* proteins and virulence.

## Background

In spite of the huge efforts to overcome the burden of tuberculosis (TB), nearly 10 million incident cases of TB cases, the death of 1.1 million HIV–negative TB patients and an additional 0.35 million deaths from HIV–associated TB are reported each year. Unfortunately, the selection and spread of multidrug–resistant (MDR) *Mycobacterium tuberculosis* strains worsen the scenario, since an estimated 0.65 million cases of MDR–TB were documented for the year 2010 [1]. Clearly, in addition to the improvement of human population welfare, the development of new vaccines, early diagnosis tests, and pharmacological treatments, a precise knowledge on mycobacteria–host cell interactions is also a requirement for the successful control of TB.

In this regard, some bacterial pathogenicity factors have been shown to contain N–terminal mitochondrial targeting signals [2,3] and a diverse array of bacterial proteins including some bacterial toxins from enteropathogenic *E.coli. Salmonella spp, N. gonorrhoea, N. meningitides, A. baumanii, H. pylori, S. aureus, S. pneumoniae, C. sordelli,* and *C. difficile* have been shown to target mitochondria [4].

Two proteins of particular interest are mitochondrial–associated protein (Map, former Orf19) and EspF from enteropathogenic *E.coli*, which enter the host cell via the type 3 secretion system and colocalize with host mitochondria [2]. It is worth noting that, Map causes alterations, in the form of mitochondrial membrane potential (Δψ_m_) dissipation but apparently it is not responsible for enteropathogenic *E.coli*–induced host cell apoptosis [2,4,5].

Mycobacterial infection affects mitochondrial function [6-8] and it has been suggested that Δψ_m_ changes are related to the mycobacterial strain′s virulence [7,8]. However, the identity of the molecules responsible for such a response has not been defined. Recently, the *M. tuberculosis* protein PE_PGRS33 was shown to localize within host cell mitochondria, and in doing so, induces host cell apoptosis [9].

Since mitochondria targeting proteins such as Map and EspF (from enteropathogenic *E. coli*) are secreted proteins, we thought that it is likely that at least some of the mycobacterial proteins that potentially target host cell mitochondria are also secreted proteins. Therefore, we set up a bioinformatic search of *M. tuberculosis* proteins likely to be secreted and to target host cell mitochondria.

The *M. tuberculosis* genome was first made available in 1998, opening the possibility for in–depth analysis of the possible pathogenic mechanisms involved in the course of mycobacterial infections [10]. Other mycobacterial genomes have been elucidated since, and new bio–informatic resources allow for *in silico* analysis of their genomes [11,12].

Genome wide analysis predicts that *M. tuberculosis* H37Rv and CDC1551 contain 3,924 and 4,246 genes, respectively [10,11].

By using freely available data bases and bioinformatic tools, we were able to single out 19 *M. tuberculosis* CDC1551 predicted proteins as likely candidates for targeting mitochondria.

Among these, five PE/PE_PGRS family proteins deserve particular attention. About 10% of the potential coding capacity of *M. tuberculosis* accounts for two large unrelated gene families encoding the PE and PPE proteins. The names PE and PPE are derived from the motifs Pro–Glu (PE) and Pro–Pro–Glu (PPE), and the largest class of the PE family in *M. tuberculosis* H37Rv is the PE_PGRS subfamily which consist of proteins with a PE domain followed by a C–terminal glycine–rich extension encoded by the PGRS motif (polymorphic GC–rich repetitive sequence) [13,14]. As a family, the PE/PE_PGRS proteins are polymorphic [10] and account for many of the differences found between the avirulent (H37Ra) and virulent (H37Rv) strains of *M. tuberculosis* and, in consequence, have been proposed as possible virulence factors [15].

## Results

### *M. tuberculosis* proteins predicted to be secreted and to target host cell mitochondria

**Figure 1:**
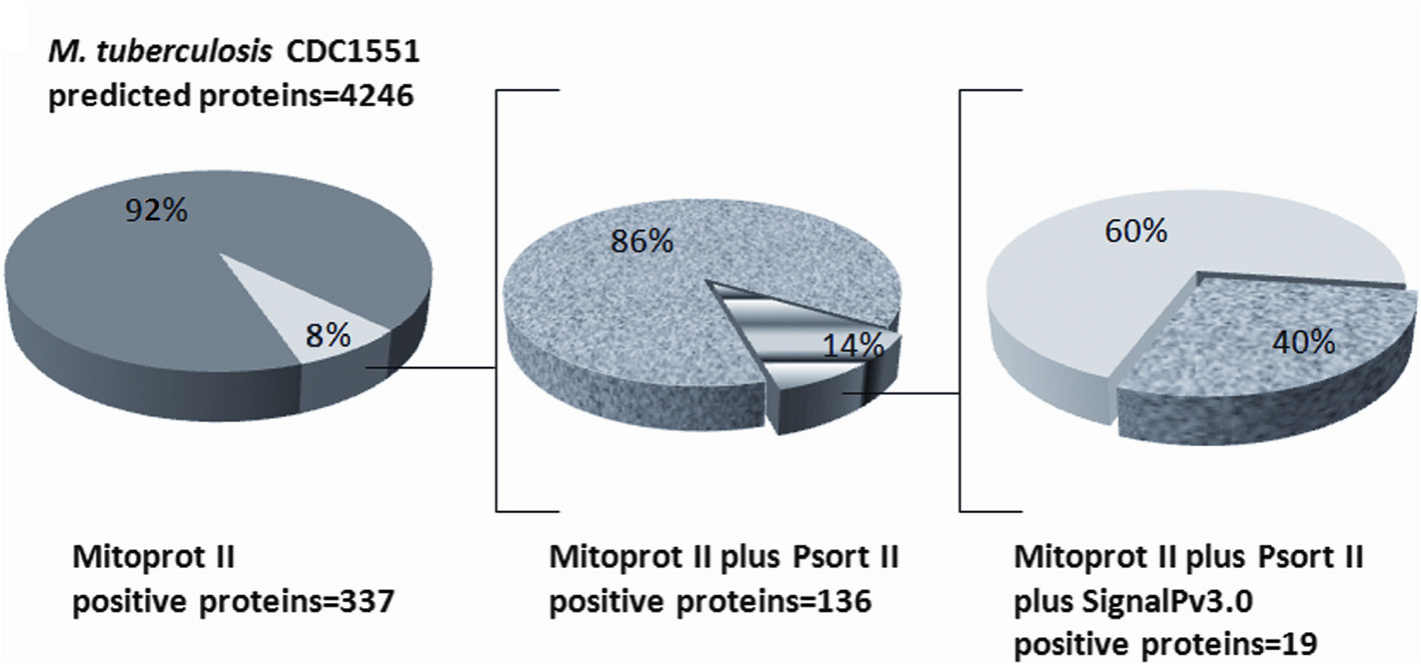
**Distribution of predicted *M. tuberculosis *CDC1551 proteins according to their signal peptides.** The 4, 246 predicted proteins of *M. tuberculosis* CDC1551 were analyzed for the presence of mitochondrial targeting sequences by the predictive algorithm MitoProt II, the 337 proteins that scored positive were analyzed by PSORT II, rendering 136 proteins likely to target mitochondria. Finally, these 136 proteins were analyzed for the presence of secretory signal peptides by the SignalP software.

The whole 4,246 predicted proteins from the *M. tuberculosis* CDC1551 genome as shown in the JCVI/CMR webpage were analyzed for the presence of mitochondrial targeting sequences by using the MitoProt II–v1.101 algorithm. Although previous analyses using this algorithm on other bacterial proteomes have set the probability limit at 0.700 to consider a protein as likely to target mitochondria [16], we decided for a more stringent analysis and therefore we set the probability limit at 0.8500 that would help to reduce false negatives. As shown in Figure 1, a total of 337 out of 4,246 *M. tuberculosis* CDC1531 proteins are predicted to have mitochondrial targeting potential. Next we analyzed those 337 proteins by using another mitochondrial targeting predictive algorithm (PSORT II), and found that 136 out of the 337 proteins scored as likely to target host cell mitochondria. Finally, the 136 predicted proteins that scored positive for both predictive algorithms were further analyzed for the probability of harboring secretory signal peptides by using the SignalP 3.0 software. Taken together, these three bioinformatic resources allowed us to identify 19 predicted proteins with the probability of harboring secretory peptide signals as well as mitochondrial targeting signals. Table 1, lists the identity of the 47 *M. tuberculosis* predicted proteins (Top–47) with the highest combined MitoProt II and PSORT II probability of targeting mitochondria. Table 2, list the 19 predicted proteins from *M. tuberculosis* CDC1551strain, likely to be secreted by the mycobacteria and to target host cell mitochondria.

**Table 1:** **Top–47 scored *M. tuberculosis *CDC1551 proteins likely to target mitochondria**

**CDC1551 LOCUS NAME**	**MITOPROT II SCORE**	**PSORT II SCORE**	**PUTATIVE IDENTIFICATION**
MT_0498	0,9908	0,9570	carbon–nitrogen hydrolase family protein
MT_2329	0,9956	0,9130	hypothetical protein
MT_40401	0,9717	0,9130	conserved hypothetical protein
MT_3169	0,9998	0,8700	bah acetyl l–hydrolase
MT_1950	0,9988	0,8700	conserved hypothetical prorein
MT_1950	0,9988	0,8700	MťM–related protein
MT_3682	0,9904	0,8700	conserved hypothetical protein
MT_1161	0,9895	0,8700	DNA–binding protein, putative
MT_3067	0,9884	0,8700	transcriptional regulator, lc lR family
MT_2080	0,9871	0,8700	conserved hypothetical protein
MT_3357	0,9925	0,8260	hypothetical protein
MT_0118	0,9796	0,8260	PE_PGRS family protein
MT_0503	0,9348	0,8700	MagC–related protein
MT_1260	0,9965	0,7830	hypothetical protein
MT_3204	0,9956	0,7830	hypothetical protein
MT_23652	0,9932	0,7830	hypothetical protein
MT_0644	0,9028	0,8700	hypothetical protein
MT_35421	0,9887	0,7830	hypothetical protein
MT_1210	0,9318	0,8260	conserved hypothetical protein
MT_1284	0,9264	0,8260	hypothetical protein
MT_23301	0,9603	0,7830	hypothetical protein
MT_2838	0,9987	0,7390	PPE family protein
MT_1457	0,9983	0,7390	hypothetical protein
MT_1866	0,9983	0,7390	PE_PGRS family protein
MT_0903	0,9983	0,7390	hypothetical protein
MT_3166	0,8710	0,8260	hypothetical protein
MT_07241	0,9573	0,7390	hypothetical protein
MT_0745	0,9982	0,6960	rplR ribosomal protein L18
MT_39721	0,9551	0,7390	hypothetical protein
MT_2954	0,9967	0,6960	lS1539, resolvase
MT_3026	0,9960	0,6960	mythyltransferase, putative
MT_0443	0,9906	0,6960	hypothetical protein
MT_0144	0,9892	0,6960	p450 heme–thiolate protein
MT_07621	0,9887	0,6960	hypothetical protein
MT_32201	0,9434	0,7390	hypothetical protein
MT_0572	0,9834	0,6960	oxidoreductase, short–chain dehydrogenase/reductase family

**Table 2:** ***M. tuberculosis *CDC1551 proteins predicted to be secreted and targeted to host cell mitochondria**

**CDC1551**	**PUTATIVE IDENTITY**
MT_0068	mitR protein
MT_0118	virulence factor mce family protein
MT_0349	PE family protein
MT_1548	Conserved hypothetical protein
MT_0824	Hypothetical protein
MT_0854	PE_PGRS family protein
MT_1105	Hypothetical protein
MT_1210	Conserved hypothetical protein
MT_1216	Hypothetical protein
MT_1934	Hypothetical protein
MT_1455	lipoprotein, 27kDA
MT_1866	PE_PGRS family protein
MT_1950	Conserved hypothetical protein
MT_2662	bacterial extracellular, solute–binding protein
MT_2690	PE–PGRS family protein
MT_3169	bac acetyl–hydrolase
MT_3367	Conserved hypothetical protein
MT_2662	bacterial extracellular, solute–binding protein
MT_2690	PE–PGRS family protein
MT_3169	bah acetyl–hydrolase
MT_3367	Conserved hypothetical protein
MT_3413	Hypothetical protein

### PE and PE_PGRS family proteins are among the *M. tuberculosis* CDC1551 proteins likely to be secreted and targeted to host cell mitochondria

Table 2 shows that the bioinformatic procedure used in this work selected 19 out of 4,246 predicted proteins from the *M. tuberculosis* CDC1551 strain genome that are predicted to be secreted and targeted to the host cell mitochondria. By looking at the annotated characteristics of those proteins, it is evident that members from the PE and PE_PGRS family proteins are overrepresented (5/19, 26.3%), since the total number of PE and PE_PGRS proteins is estimated to be 94, by manually counting them from the JCVI/CMR *M. tuberculosis* CDC1551 list (94/4,246, 2.21%). Interestingly, a recent comparative analysis among mycobacterial genomes, performed in an attempt to identify the mutations that lead to the loss of virulence in the *M. tuberculosis* H37Ra strain, showed that most mutations were confined to the PE/PPE/PE_PGRS family genes, highlighting the likely role of those proteins as virulent factors [15]. Accordingly, we performed a search for homologous proteins in *M. tuberculosis* H37Ra, *M. tuberculosis* H37Rv, and *M. bovis* BCG Pasteur 1173P2 strain and then analyzed those homologous proteins by using MitoProt II, PSORT II, and SignalP algorithms as previously described. Table 3 shows the protein codes for each mycobacterial strain, as well as the possible identity and the scores for the three aforementioned predictive algorithms. These analyses showed that the percentage of similarity among the identified *M. tuberculosis* CDC1551 strain PE or PE_PGRS proteins and their H37Ra, H37Rv, and BCG Pasteur strain homologous proteins ranged from 53.4% to 81.9%. The H37Ra (MRA_0115) and BCG (BCG_0142) homologous to CDC1551 MT_0118 protein are predicted as not mitochondrial targeted, whereas the H37Rv (Rv0109) scored positive just as well as MT_0118, and the same holds true for the three homologous proteins to MT_0349, but none of them seem to have secretory signal sequences. All six homologous proteins to CDC1551 MT_0854 and MT_1866 did not score positive for mitochondrial targeting sequences. On the other hand, the three homologous proteins for CDC1551 MT_2690 are predicted to harbor mitochondrial targeting sequences. With the exception of MRA_0344, Rv0335c, and BCG_0374, all other homologous proteins are predicted to harbor secretory signal sequences.

**Table 3:** **Comparative analysis of some *M. tuberculosis *CDC1551 strain PE and PE_PGRS proteins with their H37Ra, H37Rv and BCG Pasteur strain orthologes**

	**CDC 1551**	**H37Ra**	**H3TRv**	**BCG Pasterur 1173P2**
	**MT_0118**	MRA_0115	Rv0109	BCG_0142
Putative identity	PE–PGRS family protein			
% Similarity		81.4	81.4	81.4
MITOPROT II				
Score	0.9796	0.1144	0.9799	0.1144
PSORT II Score	82.6%	11.1%	82.6%	11.1%
Signal P Score	0.988	0.999	0.998	0.999
	**MT_0349**	MRA_0344	Rv0335c	BCG_0374
Putative identity	PE family protein			
% Similarity		81.9%	81.9%	81.3%
MITOPROT II				
Score	0.9902	0.0157	0.9746	0.0155
PSORT II Score	73.9%	17.4%	69.6%	21.7%
Signal P Score	0.640	NS	NS	NS
	**MT_0854**	MRA_0841	Rv0832	BCG_0885
Putative identity	PE–PGRS family protein			
% Similarity		56.2%	56.2%	56.2%
MITOPROT II				
Score	0.9326	0.4901	0.5935	0.5738
PSORT II Score	73.9%	4.3%	8.7%	11.1%
Signal P Score	0.999	0.998	0.993	0.998
	**MT_1886**	MRA_1830	Rv1818c	BCG_1853c
Putative identity	PE–PGRS Family			
% Similarity		53.4%	53.4%	53.4%
MITOPROT II				
Score	0.9983	0.1494	0.1494	0.1475
PSORT II Score	73.9%	13.0%	13.0%	13.0%
Signal P Score	0.974	0.991	0.991	0.991
	**MT_2690**	MRA_2643	Rv2615c	BCG_1035c
Putative identity	PE–PGRS Family			
% Similarity		79.0%	79.0%	78.2%
MITOPROT II				
Score	0.9472	0.9472	0.8178	0.9488
PSORT II Score	52.2%	52.2%	13.0%	52.2%
Signal P Score	0.917	0.917	0.849	0.917
NS non signal				

### Rv1411c (p27), and Rv1818c (PE_PGRS33) *M. tuberculosis* proteins target host cell mitochondria

Based on the list of 19 *M. tuberculosis* CDC1551 proteins likely to target host cell mitochondria (Table 2) as well as on the H37Ra, H37Rv, and BCG Pasteur 1173P2 homologous proteins to *M. tuberculosis* CDC1551 (Table 3), we selected four proteins to be experimentally tested for targeting mitochondria: MT_1866, and its H37Rv ortholog Rv1818c (PE_PGRS33); the H37Rv ortholog of MT_0118 (Rv0109 or PE_PGRS1); and the H37Rv ortholog of MT_1455 (Rv1411c or p27). In addition, the *M. tuberculosis* Rv2031c (α–crystallin) protein that scored negative for mitochondria targeting sequence in a Mitoprot II and PSORT II query, was used as a negative control. Rv1818c has recently shown to target mitochondria [9] and therefore served the purpose of a positive control. All *M. tuberculosis* recombinant proteins harbour a histidine tail that allows detection by means of a FITC–labelled anti–His antibody, as described in methods.

**Figure 2:**
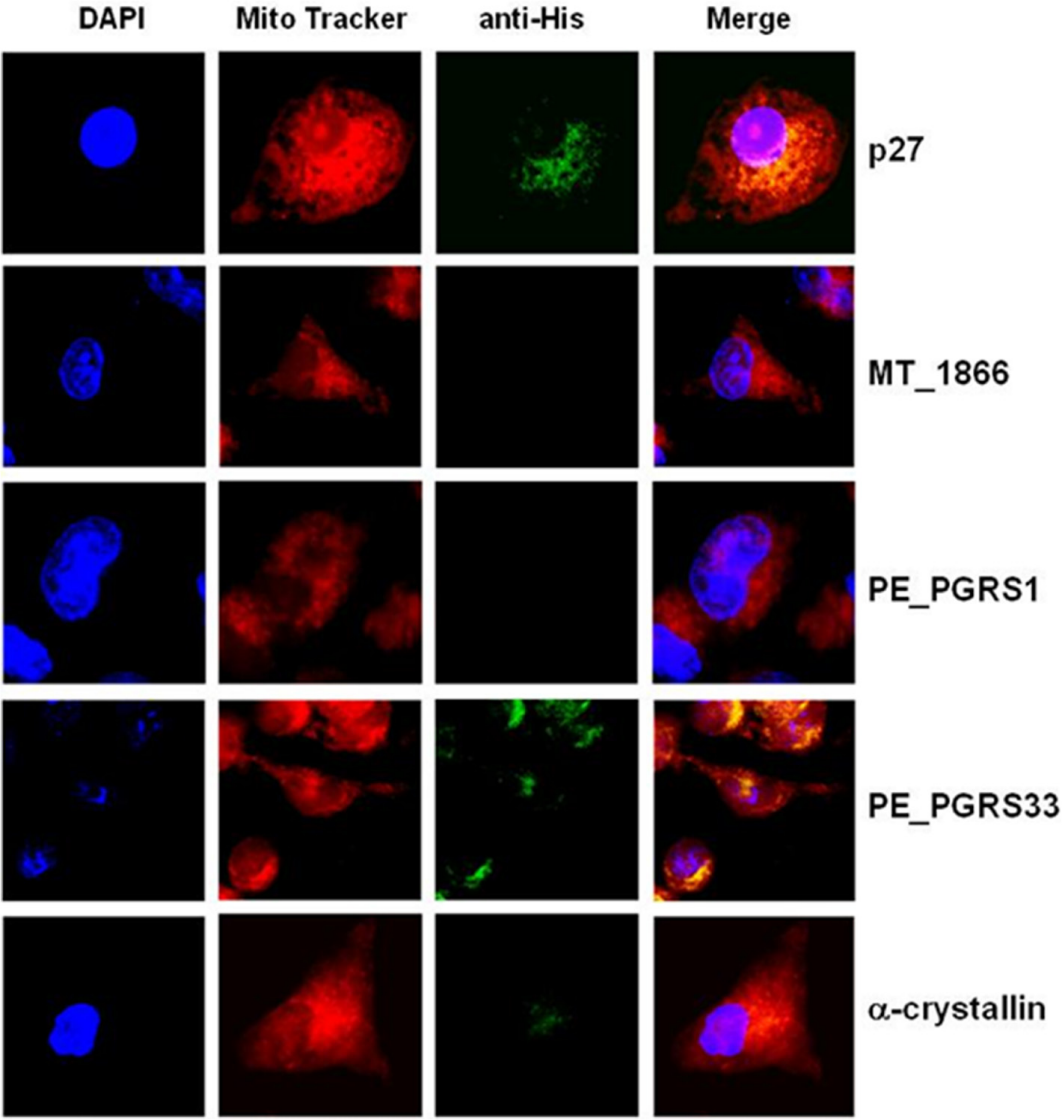
***M. tuberculosis *p27 and PE_PGRS33 target J774 cell mitochondria.** J774 macrophages exposed for 2 h to 1 μg/ml of p27, MT_1866, PE_PGRS1, PE_PGRS33, or α–crystallin His–tagged recombinant proteins were labelled with Mito Tracker red CMX ROS (mitochondria, red), FITC–labelled anti His antibody (recombinant protein, green), and DAPI (nuclei, blue), and then analyzed by confocal microscopy, in order to assess targeting of recombinant proteins to host cell mitochondria. Results are representative of multiple microscope fields form three independent experiments.

Figure 2 shows that p27, and PE_PGRS33 colocalize with mitochondria in J774 cells cultured for 2 h in the presence of 1 μg/ml of the correspondent recombinant protein. MT_1866 and PE_PGRS1 do not seem to colocalize with mitochondria, whereas α–crystallin protein, as expected, do not colacalize with mitochondria.

## Discussion

Macrophages are the main host cells for *M. tuberculosis*, therefore considerable attention has been devoted to the analysis of the mycobacteria–containing phagosomes [17] and the way by which mycobacteria inhibit their association with lysosomes [18-20], and more recently the host–pathogen cross–talk by transcriptional analyses has gained interest [21,22].

On the other hand, it has been shown that *M. tuberculosis* infection controls host cells, either by inducing [23,24] or inhibiting their death [25,26], and some *M. tuberculosis*–derived molecules have been associated with those opposing biological activities [27-29].

Mitochondria play a pivotal role in programmed cell death [30], and mitochondrial activity has been shown to be disrupted by mycobacterial infection in a process that appears to be dependent on the strain′s virulence [6-8].

It has been shown that several secreted microbial proteins are transferred to the cell during infection and may target mitochondria, playing an important role in bacterial pathogenicity [4,31]; and more recent observations suggest that the mitochondrial role in the regulation of cellular responses to pathogens may go further than the sole regulation of host cell death [32].

Taking all this into account we wanted to analyze the *M. tuberculosis* genome and their predicted proteome in search of moieties likely to play a mitochondrial–mediated pathogenicity role, by using bioinformatic tools.

Gomez et al. [33] have already identified *M. tuberculosis* secreted proteins by performing a bioinformatic search on the 3,924 initially predicted proteins from the *M. tuberculosis* H37Rv genome [10].

Here, we used a different approach. The whole 4,246 predicted proteins from the *M. tuberculosis* CDC1551 genome [11] were first analyzed for the presence of mitochondrial targeting sequences by using MitoProt II–v1.101 and PSORT II algorithms. The CDC1551 strain was chosen on the basis of it being isolated from a relatively recent tuberculosis outbreak.

The MitoProt II analysis we performed was more stringent than similar searches on other bacterial proteomes were authors concluded that at least 5% of proteins from *E.coli* are predisposed for targeting mitochondria [16]. In this work, by using two predictive algorithms, it was found that, in *M. tuberculosis* CDC1551, 136 out of 4,246 (3.2%) proteins are likely to target mitochondria. Mitoprot II–V1.101 has a level of accuracy of 94.76%–97.54% for successfully predicting mitochondrial proteins and 76.79%–87.92% for successfully predicting non–mitochondrial proteins in a protein training group [34], and PSORT II has an accuracy of approximately 60%–86% [35].

The 136 predicted proteins likely to target mitochondria were further analyzed for the presence of secretory signal peptides by using the SignalP 3.0 hidden Markov model, which, incidentally, has recently been reported to outperform other similar tools in predicting *M. tuberculosis* secreted proteins with a level of accuracy of 100%, as it predicted no false positives or false negatives in a negative set (n=61), and in a positive set (n=57) of mycobacterial proteins, respectively. In addition, this algorithm showed 78.9% accuracy in predicting the actual cleavage site [36]. Their analysis predicted that between 7.8% and 10.5% of the proteins in the proteomes of different mycobacteria (including the CDC1551 strain) are secreted proteins. Our SignalP v3.00 hidden Markov model search predicted that 19 out of the 136 proteins likely to target mitochondria are also secreted proteins, i.e., 13.9%.

In their analysis, Gomez et al. [33] excluded 47 PE/PPE proteins out of the top *M. tuberculosis* 208 proteins predicted to be secretory, arguing that biased amino acid composition and a repetitive primary sequence could lead to unreliable results in sequence analyses. We did not exclude these proteins from our analysis and we found that some PE_PGRS proteins are not predicted to harbour mitochondrial targeting or secretory signals (data not shown); thus, suggesting that the amino acid composition of these proteins did not introduce any bias in our bioinformatic output. In addition, it has recently been shown that some PE_PGRS and PPE proteins are secreted by at least one mycobacterial species (*M. marinum*) and that those proteins are transported via the type VII secretion system ESX–5 [37].

Interestingly, among the 19 CDC1551 proteins likely to be secreted and to targeted mitochondria, 5 belong to the PE/PE_PGRS protein family (Table 2).

About 10% of the coding capacity of the genome is devoted to two large unrelated families of acidic, glycine–rich proteins, the PE (Pro–Glu) and PPE (Pro–Pro–Glu) families, often based on multiple copies of the polymorphic repetitive sequence referred to as PGRSs (polymorphic GC–rich repetitive sequences). It has been suggested that these protein families could be of immunological importance [10], and a possible source of antigenic variability that would help *M. tuberculosis* to evade the host immune system during infection [14]. In addition, by using a computational strategy based on phylogenetic profiling and comparative proteomic analysis, Meszaros et al. [38] have proposed that *M. tuberculosis* PE/PPE proteins can be considered as potential targets for drug design. Although the biological function of the PE proteins is still under study, the PE_PGRS member annotated as Rv1759c has been characterized as fibronectin–binding protein [39] and a genetic screen of *M. bovis* BCG Pasteur mutagenized with the transposon Tn5367, identified a gene, identical to the Rv1818c gene of *M. tuberculosis*, encoding a PE_PGRS protein that influences the interactions of mycobacteria with macrophages [40]. Rv1818c has also been implied in T lymphocytes apoptosis [41], and it has been shown that most genetic differences between *M. tuberculosis* H37Rv and H37Ra strains, likely to function as virulence factors are in the PE/PPE/PE_PGRS family genes [15].

The availability of suitable bioinformatic tools allowed us to single out 19 M. tuberculosis proteins as likely to be secreted and to target host cell mitochondria, among them five PE/PE_PGRS family protein members. Recently, Cadieux et al. [9] showed that the *M. tuberculosis* PE_PGRS33 protein (encoded by the Rv1818c gene) co–localize with mitochondria when expressed in human rhabdomyosarcoma (RD) cells, and mitochondrial localization of PE_PGRS33 protein was followed by induction of cell death [9]. This finding is somewhat in contrast with our *in silico* analysis, since PE_PGRS33 (RV1818c) is not predicted to harbour a mitochondrial targeting sequence by MitoProt or PSORT II (Table 3) Moreover, the *M. tuberculosis* CDC1551 protein MT_1866 which is the ortholog of Rv1818c is predicted to harbour a mitochondria targeting sequence (Table 3) and experimentally failed to do so (Figure 2). A possible explanation for this is that PE_PGRS proteins are unreliably alignable or differences in the annotation for the strain CDC1551 as compared for the H37Rv stain of *M. tuberculosis*. In addition the similarity score between RV1818c and MT_1866 is just 53.4%, a very low score for considering true homology between these two proteins (Table 3).

All in all, our results show, in another experimental system (J774 murine macropheges exposed to the recombinant protein), that PE_PGRS33 targets host cell mitochondria, thus confirming Cadieux et al. [9] findings.

In addition, based on bioinformatic analyses, we present evidence that Rv1411c (p27) which is the *M. tuberculosis* H37Rv ortholog of *M. tuberculosis* CDC1551 MT_1455 and a known virulence factor, targets mitochondria (Figure 2).

Identifying other *M. tuberculosis* proteins that target mitochondria would help to clarify the structural requirements for mitochondrial targeting and more important, the role of those proteins in the *M. tuberculosis*–host cell communication. This work provided a list of 19 candidate proteins, and after experimentally testing some of them and identifying p27 as a mitochondria targeting protein, still leaves some candidate proteins for further analysis.

## Conclusions

Over the past decade it has been shown that in addition to their role in ATP production, mitochondria also function as signal transducers platforms and have an essential role in immune responses and host–pathogen interactions.

In analysing the pathogen–host cell crosstalk, it has been shown that several pathogen proteins target mitochondria thus controlling the host cell fate. Here, we conducted a bioinformatic analysis aimed at identifying *M. tuberculosis* proteins that, based on aminoacid sequence, could possible be secretory, and target mitochondria.

Our results singled out 19 out of 4, 246 *M. tuberculosis* predicted proteins as likely to be secretory and harbour mitochondrial targeting sequences. Interestingly, several proteins considered to be virulence factors are included and over–represented in this 19 protein list. Four such proteins were experimentally tested and while confirming that PE_PGRS33 targets mitochondria, a new *M. tuberculosis* mitochondria targeting protein was identified (p27).

This work suggests a correlation between *M. tuberculosis* protein mitochondrial targeting potential and virulence. The interaction between *M. tuberculosis* and its host cell is complex and, as we think, understanding the role of mitochondria in mycobacterial infections and the role of *M. tuberculosis* specific proteins in the crosstalk with host cells at the level of mitochondria will lead us to a better explanation of phenomena such as tuberculosis latency.

## Methods

### Bioinformatic analysis of predicted *M. tuberculosis* proteins

*M. tuberculosis* predicted protein sequences were downloaded from the *M. tuberculosis* CDC1551 Genome Project from J. Craig Venter Institute/Comprehensive Microbial Resource (JCVI/CMR version 1.0) ( http://cmr.jcvi.org/tigr–scripts/CMR/GenomePage.cgi?database=gmt).

Protein sequences were then analyzed by using the MitoProt II–v1.101 Software ( http://ihg2.helmholtz–muenchen.de/ihg/mitoprot.html), which calculates the N–terminal protein region that can support a mitochondrial targeting sequence and the cleavage site, as described [34]. *M. tuberculosis* putative proteins with a probability of at least 0.8500 of having a mitochondrial targeting sequence were selected and re–analyzed by means of the PSORT II prediction algorithm for subcellular localization ( http://psort.ims.u–tokyo.ac.jp/form2.html) which uses the k nearest neighbours classifier [35]. Those proteins that in addition of scoring well for MitoProt II had the highest probability of being targeted to mitochondria by PSORT II were further analyzed for secretory signal peptides by using SignalP 3.0 ( http://www.cbs.dtu.dk/services/SignalP/), which predicts the presence and location of signal peptide cleavage sites in amino acid sequences from Gram–positive prokaryotes, Gram–negative prokaryotes, and eukaryotes. The method incorporates a prediction of cleavage sites and a signal peptide/non–signal peptide prediction based on a combination of several artificial neural networks and hidden Markov models, as described [42-44].

The amino acid sequences of some proteins likely to harbor mitochondrial targeting sequences, and to be secreted by the CDC1551 strain of *M. tuberculosis*, were compared against the homologous proteins of *M. tuberculosis* H37Rv, *M. tuberculosis* H37Ra, and *M. bovis* BCG Pasteur 1173P2 strain, by using the “Protein vs. All Alignment” tool at the JCVI/CMR webpage. The retrieved amino acid sequences were then analyzed by MitoProt II–v1.101, PSORT II, and SignalP 3.0 algorithms as previously described.

### *M. tuberculosis* recombinant proteins

The cloning, expression and purification of the *M. tuberculosis* Rv1411c (p27) protein was performed as previously described [45]. The full length of Rv1818c (PE_PGRS33) gene cloned into PET15b fused to a histidine tag was a kind gift from Dr. M.J. Brennan (CBER, FDA, Bethesda, MD, USA). The coding regions for the mycobacterial antigens were amplified by PCR with the high fidelity DNA polymerase *Pfx* (Invitrogen) from *M. tuberculosis* H37Rv genomic DNA (Rv1411c or p27; Rv0109 or PE_PGRS1; Rv1818c or PE_PGRS33) or *M. tuberculosis* CDC1551 (MT_1866) with specific oligonucleotide primers. PCR products were ligated into the pCR4 Blunt–TOPO vector (Invitrogen), and amplified in JM110 *Escherichia coli* strain (Stratagene) and then subcloned into pET15b vector, that was used for the transformation of the *E. coli* strains Rosetta (DE3) (Novagen) or C41 (DE3) (Avidis S.A.). Expression of the proteins was induced in logarithmic phase cultures. Bacterial pellets were suspended in PBS, sonicated and centrifuged. His–tagged recombinant proteins were purified in an AKTA FLPC system (GE Healthcare), using 1 ml Histrap columns (GE Healthcare). Pooled purified recombinant proteins were dialized against 0.3 M NaCl, 20 mM NaH_2_PO_4_, pH 8.0. In addition, the *M. tuberculosis* Rv2031 protein (also known as 16 kDa antigen, hsp 16.3, hspX, and α–crystallin), obtained from E. coli and purified by histidin–affinity chromatography was used as a negative control on the basis of its low probability score for targeting mitochondria (Mi MitoProt II–v1.101, and PSORT II). In all cases, protein concentration was quantified and recombinant proteins were stored at −70°C until use.

### Mitochondrial targeting of *M. tuberculosis* recombinant proteins

J774.A1 murine macrophages were plated on glass cover–slips in 6–well culture plates (Corning) at a density of 5×10^5^ cells/well and incubated overnight at 37°C in 5% CO_2_ atmosphere. After washing, cells were incubated in the presence of the indicated *M. tuberculosis* recombinant proteins (p27, MT_1866. PE_PGRS1, PE_PGRS33, or α–crystallin used as a negative control) for 2 h. Cells were then labelled with MitoTracker red CMXRos (Invitrogen) at a final concentration of 100 nM, for 30 min and subsequently fixed and permeabilized (Cytofix/Cytoperm, BD Biosciences) and stained with FITC–labelled anti–His antibody (Miltenyi Biotec). After further washing, cells were mounted in DAPI–containing Vectashield (Vector). Imaging was performed in a LSM5 Pascal (Zeiss) confocal microscope, using a 100x oil immersion objective. Online data acquisition and further fluorescence analysis were carried out using LSM5 image browser (Zeiss).

## Abbreviations

*M. tuberculosis*: *Mycobacterium tuberculosis*; PE: Prolin–Glutamic acid motif; PPE: Prolin–Prolin–Glutamic acid motif; PGRS: Polymorphic GC–rich repetitive sequence.

## Competing interest

The authors declare no competing interest.

## Authors’ contributions

MMBMA conceived the idea, experimental design,analyzed data; ISPG performed experiments; CE provided special reagents, experimental design, analyzed data; MSM performed experiments; RHP provided special reagents, analyzed data; FJSG conceived the idea, experimental design, performed experiments, analyzed data, and wrote the paper. All authors read and approved the final manuscript.
